# Twin birth changes DNA methylation of subsequent siblings

**DOI:** 10.1038/s41598-017-08595-6

**Published:** 2017-08-16

**Authors:** Shuai Li, Eunae Kim, Ee Ming Wong, Ji-Hoon Eric Joo, Tuong L. Nguyen, Jennifer Stone, Yun-Mi Song, Louisa B. Flander, Richard Saffery, Graham G. Giles, Melissa C. Southey, Joohon Sung, John L. Hopper

**Affiliations:** 1 Centre for Epidemiology and Biostatistics, Melbourne School of Population and Global Health, The University of Melbourne, Parkville, Victoria, Australia; 2 Department of Epidemiology, School of Public Health, Seoul National University, Seoul, South Korea; 3 Genetic Epidemiology Laboratory, Department of Pathology, University of Melbourne, Parkville, Victoria, Australia; 4 Centre for Genetic Origins of Health and Disease, Curtin University and the University of Western Australia, Perth, Western Australia Australia; 5 Department of Family Medicine, Samsung Medical Center, Sungkyunkwan University School of Medicine, Seoul, South Korea; 6 Murdoch Childrens Research Institute, Royal Children’s Hospital, Parkville, Victoria, Australia; 7 Department of Paediatrics, University of Melbourne, Parkville, Victoria, Australia; 8 Cancer Epidemiology Centre, Cancer Council Victoria, Melbourne, Victoria, Australia; 9 Institute of Health and Environment, Seoul National University, Seoul, South Korea

## Abstract

We asked if twin birth influences the DNA methylation of subsequent siblings. We measured whole blood methylation using the HumanMethylation450 array for siblings from two twin and family studies in Australia and Korea. We compared the means and correlations in methylation between pairs of siblings born before a twin birth (BT siblings), born on either side of a twin birth (B/AT pairs) and born after a twin birth (AT siblings). For the genome-wide average DNA methylation, the correlation for AT pairs (r_AT_) was larger than the correlation for BT pairs (r_BT_) in both studies, and from the meta-analysis, r_AT_ = 0.46 (95% CI: 0.26, 0.63) and r_BT_ = −0.003 (95% CI: −0.30, 0.29) (*P* = 0.02). B/AT pairs were not correlated (from the meta-analysis r_BAT_ = 0.08; 95% CI: −0.31, 0.45). Similar results were found for the average methylation of several genomic regions, e.g., CpG shelf and gene body. BT and AT pairs were differentially correlated in methylation for 15 probes (all *P* < 10^−7^), and the top 152 differentially correlated probes (at *P* < 10^−4^) were enriched in cell signalling and breast cancer regulation pathways. Our observations are consistent with a twin birth changing the intrauterine environment such that siblings both born after a twin birth are correlated in DNA methylation.

## Introduction

DNA methylation mainly occurs at cytosine-guanine dinucleotides (CpG) sites through the conversion of cytosine to 5-methylcytosine. This is one of several epigenetic modifications known to be involved in transcription regulation without changing the DNA sequence. DNA methylation has been proposed to play a critical role in the etiology of complex traits and diseases^1^.

Enlarged by pregnancy and then undergoing involution, the uterus potentially acquires specific changes as a consequence of previous births. Multiple pregnancy, an atypical pregnancy, causes even greater changes to the intrauterine environment as the uterus is enlarged far more than with a singleton pregnancy. For example, using the fundal height of women with normal singleton pregnancy as a measure of uterine distension, 99% women with twin pregnancy had fundal height > 2 standard deviations larger than the average of women with singleton pregnancy at the same gestation week^2^. Multiple pregnancy is also known to change maternal physiology^3, 4^ and increase the risks of several maternal complications^4, 5^, such as gestational diabetes and preeclampsia. In fact, many studies have found evidence that prenatal factors or intrauterine environment play a key role in shaping the DNA methylation of offspring^6–11^, with further evidence that some of these effects tend to be long-lasting and persist into later life^10–13^. In light of this we asked whether twin birth can alter the intrauterine environment in such a manner that influences the methylation status of subsequent siblings such that persons born after a twin birth have different methylation features compared with those born before a twin birth.

To answer this question, we studied the different methylation measures, including the global methylation, the average methylation of genomic regions and methylation at individual site, of middle-aged siblings from two twin and family studies: the Australian Mammographic Density Twins and Sisters Study (AMDTSS) and the Korean Healthy Twin Study (KHTS). We compared the means of different methylation measures between siblings born before a twin birth (denoted as BT siblings) and siblings born after a twin birth (denoted as AT siblings). We also compared the correlations across three types of sibling pairs: BT pairs, AT pairs and pairs born either side of a twin birth (B/AT pairs).

## Results

### Characteristics of included subjects

Table 1 shows the characteristics of included subjects from the AMDTSS and KHTS. The mean age (standard deviation) of subjects was 56.6 (8.0) and 44.9 (5.6) years, respectively. BT siblings were on average 7 years older than AT siblings in the AMTDSS (*P* < 0.01). There was no evidence of the BT and AT siblings differed in any other characteristics.

**Table 1:** Characteristics of BT and AT siblings from the AMDTSS and KHTS^*^.

	AMDTSS			KHTS		
	BT siblings	AT siblings	*P*-value^†^	BT siblings	AT siblings	*P*-value^†^
N	112	103		27	15	
Age, mean ± SD	59.9 ± 7.3	52.9 ± 7.1	< 0.01	46.2 ± 6.1	42.5 ± 5.0	0.05
Sex, N (%)			—			0.96
Females	112 (100.0)	103 (100.0)		16 (59.3)	9 (60.0)	
Males	0	0		11 (40.7)	6 (40.0)	
BMI, mean ± SD	27.5 ± 5.8	26.6 ± 6.0	0.26	24.8 ± 3.0	22.8 ± 3.4	0.06
Smoking, N (%)			0.91			0.73
Never	65 (58.0)	58 (56.3)		17 (63.0)	11 (73.3)	
Ever	47 (42.0)	45 (43.7)		10 (37.0)	4 (26.7)	
Alcohol, N (%)			0.83			1.00
Never	43 (38.4)	42 (40.8)		5 (0.19)	3 (0.20)	
Ever	69 (61.6)	61 (59.2)		22 (0.81)	12 (0.80)	

^*^BT: before twin birth; AT: after twin birth; AMDTSS: Australian Mammographic Density Twins and Sisters Study; KHTS: Korean Healthy Twin Study; SD: standard deviation.

^†^ *P*-value for compare the characteristic between BT and AT siblings within each study.

### Discovery analysis

For the AMDTSS, from the comparison of means there was no evidence of a difference in GWAM, or in the average methylation of any genomic region, between BT and AT siblings (all *P* > 0.1; Table 2). From the probe-by-probe analysis, no probe differed between BT and AT siblings at the genome-wide level of significance (*P* = 10^−7^).

**Table 2:** GWAM and the average methylation of genomic regions between BT and AT siblings from the AMDTSS^*^

Genomic regions	Methylation in BT siblings^†^ (mean ± SD)	Methylation in AT siblings^†^ (mean ± SD)	*P*-value^‡^
GWAM	52.97 ± 0.32	52.99 ± 0.31	0.39
CpG island	22.77 ± 0.34	22.66 ± 0.39	0.33
CpG shelf	78.45 ± 0.47	78.58 ± 0.46	0.14
CpG shore	49.23 ± 0.43	49.23 ± 0.41	0.53
non-CGI region	74.28 ± 0.45	74.38 ± 0.44	0.19
Gene body	65.17 ± 0.35	65.22 ± 0.36	0.20
Promoter	30.83 ± 0.29	30.78 ± 0.28	0.81
Intergenic region	63.19 ± 0.40	63.23 ± 0.40	0.33
TSS1500	37.77 ± 0.34	37.76 ± 0.32	0.76
TSS200	19.40 ± 0.24	19.32 ± 0.27	0.21
5′UTR	34.00 ± 0.28	33.96 ± 0.28	0.89
1stExon	22.22 ± 0.28	22.12 ± 0.31	0.21
3′UTR	76.14 ± 0.40	76.25 ± 0.39	0.10

^*^GWAM: genome-wide average methylation; BT: before twin birth; AT: after twin birth; AMDTSS: Australian Mammographic Density Twins and Sisters Study; SD: standard deviation.

^†^Methylation is presented as the percentage of methylation, that is, beta-value × 100.

^‡^ *P*-value for compare the mean of methylation between BT and AT siblings.

**Figure 1:**
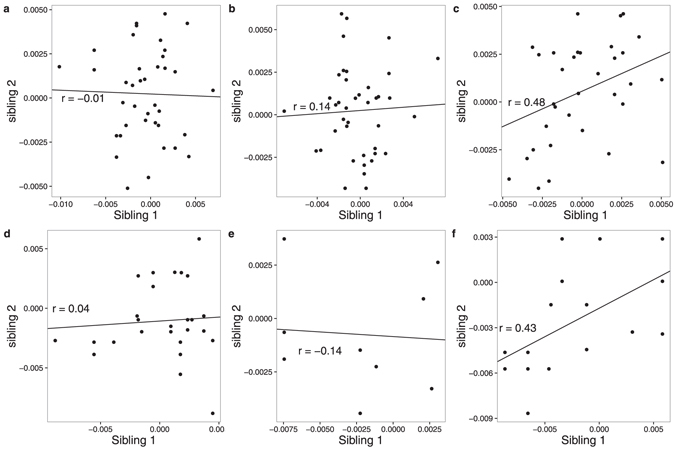
Correlations in GWAM for different types of sibling pairs in the AMDTSS and KHTS (**a**) Pairs of BT siblings in the AMDTSS. (**b**) Pairs of B/AT siblings in the AMDTSS. (**c**) Pairs of AT siblings in the AMDTSS. (**d**) Pairs of BT siblings in the KHTS. (**e**) Pairs of B/AT siblings in the KHTS. (**f**) Pairs of AT siblings in the KHTS.

Neither BT nor B/AT pairs were correlated in GWAM (r_BT_ = −0.01, 95% CI: −0.34, 0.31; r_BAT_ = 0.14; 95% CI: −0.31, 0.53) (Table 3; Fig. 1). After combining r_BT_ and r_BAT_, the estimate was 0.04 (95% CI: −0.24, 0.32). The correlation for AT pairs, however, was 0.48 (95% CI: 0.23, 0.67), larger than the correlation for BT pairs (*P* = 0.06). We did not find evidence that these correlations depended on the twins’ zygosity (*P* = 0.18), or any of the examined factors (all *P* > 0.1).

**Table 3:** Correlations in GWAM and in the average methylation of genomic regions^*^.

Genomic region	Discovery: AMDTSS				Replication: KHTS				Meta-analysis: AMDTSS + KHTS			
	r_BT_ (SE)	r_BAT_ (SE)	r_AT_ (SE)	*P* ^‡^	r_BT_ (SE)	r_BAT_ (SE)	r_AT_ (SE)	*P* ^‡^	r_BT_ (SE)	r_BAT_ (SE)	r_AT_ (SE)	*P* ^‡^
	N = 38^†^	N = 40^†^	N = 34^†^		N = 26^†^	N = 9^†^	N = 16^†^		N = 64^†^	N = 49^†^	N = 50^†^	
GWAM	−0.01 (0.17)	0.14 (0.23)	0.48 (0.15)	0.06	0.04 (0.36)	−0.14 (0.45)	0.43 (0.21)	0.35	−0.003 (0.16)	0.08 (0.21)	0.46 (0.12)	0.02
CpG island	0.001 (0.27)	0.18 (0.19)	0.23 (0.18)	0.49	−0.02 (0.25)	−0.20 (0.59)	0.36 (0.26)	0.33	−0.01 (0.19)	0.15 (0.18)	0.27 (0.15)	0.23
CpG shelf	0.15 (0.21)	−0.10 (0.36)	0.64 (0.10)	0.03	0.02 (0.30)	−0.33 (0.43)	0.44 (0.20)	0.31	0.11 (0.17)	−0.20 (0.28)	0.60 (0.09)	0.01
CpG shore	−0.08 (0.14)	0.08 (0.20)	0.29 (0.20)	0.19	0.06 (0.34)	0.01 (0.32)	0.48 (0.23)	0.31	−0.06 (0.13)	0.06 (0.17)	0.38 (0.15)	0.03
non-CGI region	0.08 (0.21)	0.11 (0.35)	0.63 (0.10)	0.02	−0.17 (0.15)	−0.39 (0.13)	0.40 (0.20)	0.15	−0.10 (0.12)	−0.34 (0.12)	0.59 (0.09)	6.2E-6
Gene body	0.02 (0.18)	0.06 (0.30)	0.56 (0.12)	0.03	0.05 (0.41)	−0.29 (0.35)	0.44 (0.20)	0.36	0.02 (0.17)	−0.09 (0.23)	0.53 (0.10)	0.01
Promoter	0.05 (0.17)	0.18 (0.16)	0.17 (0.21)	0.66	0.01 (0.27)	0.15 (0.37)	0.52 (0.21)	0.19	0.04 (0.14)	0.18 (0.14)	0.35 (0.15)	0.13
Intergenic region	−0.02 (0.20)	0.17 (0.27)	0.53 (0.13)	0.04	−0.12 (0.23)	−0.27 (0.31)	0.39 (0.21)	0.25	−0.06 (0.15)	−0.02 (0.20)	0.49 (0.11)	0.003
TSS1500	−0.03 (0.16)	0.18 (0.16)	0.21 (0.22)	0.40	0.04 (0.28)	0.15 (0.34)	0.49 (0.22)	0.24	−0.01 (0.14)	0.17 (0.15)	0.36 (0.15)	0.08
TSS200	0.36 (0.17)	0.24 (0.16)	0.17 (0.19)	0.45	−0.20 (0.14)	0.20 (0.32)	0.61 (0.19)	0.03	0.04 (0.11)	0.23 (0.14)	0.41 (0.13)	0.03
5′UTR	0.10 (0.16)	0.17 (0.17)	0.24 (0.20)	0.59	0.07 (0.29)	0.18 (0.45)	0.51 (0.21)	0.24	0.09 (0.14)	0.17 (0.16)	0.37 (0.14)	0.17
1stExon	0.27 (0.21)	0.23 (0.17)	0.13 (0.20)	0.63	−0.18 (0.18)	−0.18 (0.36)	0.51 (0.22)	0.10	0.02 (0.14)	0.16 (0.15)	0.31 (0.15)	0.14
3′UTR	0.02 (0.18)	0.05 (0.33)	0.60 (0.11)	0.01	0.20 (0.40)	−0.33 (0.50)	0.42 (0.20)	0.58	0.05 (0.16)	−0.07 (0.28)	0.56 (0.10)	0.01

^*^GWAM: genome-wide average methylation; BT: before twin birth; AT: after twin birth; BAT: born on either side of a twin birth; AMDTSS: Australian Mammographic Density Twins and Sisters Study; KHTS: Korean Healthy Twin Study; SE: standard error.

^†^Number of quasi-independent pairs.

^‡^ *P*-value for the comparison between r_BT_ and r_AT_.

For the average methylation of genomic regions, BT pairs were correlated in TSS200 (r_BT_ = 0.36; 95% CI: 0.04, 0.61) while AT pairs were not correlated (r_AT_ = 0.17; 95% CI: −0.19, 0.49); however, there was no difference between the two correlations (*P* = 0.45; Table 3). BT pairs were not correlated in any other regions (all *P* > 0.19). AT pairs were correlated in CpG shelf, non-CGI region, gene body, intergenic region and 3′UTR (all estimates > 0.5; all *P* < 6.0 × 10^−6^). For these five regions, the correlations were also different between BT and AT pairs (all *P* < 0.04). The number of statistically significant (at *P* < 0.05) comparisons, 5 out of 12, was more than the 0.6 that would have been expected by chance (*P* = 1.8 × 10^−4^). B/AT pairs were not correlated in any region (all *P* > 0.12).

From the probe analyses, for 306 the algorithm failed to converge, leaving results for 468,100 probes. There were more probes with positive correlation estimates for AT pairs compared with BT pairs (45% vs 40%, *P* < 10^−15^). The mean correlation estimate was −0.03 for BT pairs, smaller than −0.01 for AT pairs (*P* < 10^−15^). From comparing the correlations, 15 probes were identified to be differentially correlated between BT and AT pairs at the genome-wide level of significance (*P* = 10^−7^) (Table 4). Among them, two probes were more correlated for BT pairs and 13 probes were more correlated for AT pairs. This inconsistency in direction was more extreme than a 50:50 split expected under the null hypothesis that the difference is random (*P* < 0.01).

**Table 4:** Probes identified to be differentially correlated between BT and AT pairs from the AMDTSS^*^.

Probe	CHR	Position	UCSC_RefGene_Name	UCSC_RefGene_Group	Relation_to_UCSC_CpG_Island	r_BT_ (SE)	r_AT_ (SE)	*P*-value^†^
cg08757148	1	24513722	IL28RA	1stExon	Island	0.98 (0.01)	0.15 (0.11)	< 2.2E-16
cg15392109	11	118478329	PHLDB1	5′UTR	Island	−0.03 (0.18)	0.98 (0.01)	4.4E-16
cg06699489	6	158690902				−0.15 (0.12)	0.90 (0.02)	7.6E-12
cg11671265	4	78722517	CNOT6L	Body		0.07 (0.23)	0.95 (0.01)	3.2E-11
cg08410878	8	1733369	CLN8	3′UTR	Island	−0.14 (0.23)	0.93 (0.02)	3.7E-11
cg21173402	8	610095				−0.15 (0.13)	0.94 (0.02)	3.1E-10
cg05751055	6	33036504	HLA-DPA1	Body		0.93 (0.02)	−0.06 (0.11)	6.0E-10
cg19726630	3	32400704	CMTM8	Body		0.08 (0.18)	0.94 (0.02)	8.5E-10
cg14372324	20	30347798	TPX2	Body		−0.07 (0.14)	0.93 (0.02)	1.3E-09
cg07570618	1	1992389	PRKCZ	Body	S_Shore	−0.05 (0.09)	0.93 (0.02)	1.4E-09
cg00684178	2	242752080	NEU4	5′UTR	N_Shelf	−0.08 (0.12)	0.90 (0.03)	1.5E-09
cg18875674	11	73026651	ARHGEF17	Body		0.21 (0.15)	0.93 (0.02)	1.7E-09
cg06776907	1	40714009	TMCO2	Body		0.05 (0.15)	0.90 (0.02)	1.9E-09
cg22491001	13	111142037	RAB20	Body		−0.19 (0.13)	0.88 (0.03)	2.9E-09
cg19976628	3	38033516			N_Shelf	−0.06 (0.16)	0.90 (0.03)	5.7E-09

^*^BT: before twin birth; AT: after twin birth; AMDTSS: Australian Mammographic Density Twins and Sisters Study; CHR: chromosomes; SE: standard error.

^†^ *P*-value for the comparison between r_BT_ and r_AT_.

### Replication analysis

For the KHTS, neither BT nor B/AT pairs were correlated in GWAM (r_BT_ = 0.04, 95% CI: −0.58, 0.64; r_BAT_ = −0.14; 95% CI: −0.77, 0.64) (Table 3; Fig. 1). The correlation was 0.43 (95% CI: 0.05, 0.70) for AT pairs, numerically larger than r_BT_ (*P* = 0.35). We did not find evidence of sex heterogeneity in the correlations (*P* = 0.28).

For the average methylation of genomic regions, we consistently found that the AT pairs were more correlated than BT pairs between the discovery and replication analyses (*P* = 0.04; Table 3). BT pairs were not correlated in any region (all *P* > 0.16). AT pairs were correlated in all regions (all *P* < 0.05) except CpG island. The correlations for AT pairs were all numerically larger than those for BT pairs, and the difference for TSS200 was significant (*P* = 0.03). B/AT siblings were not correlated in any region except being negatively correlated in the non-CGI region (r_BAT_ = −0.39; 95% CI: −0.58, −0.17).

### Meta-analysis

From combining the AMDTSS and KHTS results, neither BT nor B/AT pairs were correlated in GWAM (r_BT_ = −0.003, 95% CI: −0.30, 0.29; r_BAT_ = 0.08; 95% CI: −0.31, 0.45). The correlation was 0.46 (95% CI: 0.26, 0.63) for AT pairs, larger than the correlation for BT pairs (*P* = 0.02; Table 3).

Neither BT nor B/AT pairs were correlated in any region, except that B/AT pairs were negatively correlated in the non-CGI region (r_BAT_ = −0.34; 95% CI: −0.53, −0.12) (Table 3). AT pairs were correlated in all regions (*P* < 0.03), except that the correlation in CpG island was marginally significant (*P* = 0.06). The correlations were different between BT and AT pairs in each of the CpG shelf, CpG shore, non-CGI region, gene body, intergenic region, TSS200 and 3′UTR regions (all *P* < 0.04).

### Pathway analysis

A total of 152 differential correlated probes (at *P* < 10^−4^), annotated to 148 unique genes, were included. The top pathways in which these probes overrepresented included “GPCR-mediated nutrient sensing in enteroendocrine cells” (4 of 85 molecules: *ADCY9*, *CASR*, *ITPR1* and *PRKCZ*), “Phospholipase C signalling” (6 of 240 molecules: *ADCY9*, *ARHGEF16*, *ARHGEF17*, *ITPR1*, *RAP1A* and *PRKCZ*), “Corticotropin releasing hormone signalling” (4 of 111 molecules: *ADCY9*, *ITPR1*, *RAP1A* and *PRKCZ*), “Clathrin-mediated endocytosis signaling” (5 of 197 molecules: *CSNK2A1*, *AP2M1*, *FGF8*, *DAB2* and *FGF1*), “Asparagine biosynthesis I” (1 of 1 molecules: *ASNS*) and “Breast cancer regulation by stathmin1” (5 of 203 molecules: *ADCY9*, *ARHGEF16*, *ARHGEF17*, *ITPR1* and *PRKCZ*; all *P* < 0.01). The top diseases and disorders included “Connective tissue disorders”, “Developmental disorder”, “Gastrointestinal disease”, “Organismal injury and abnormalities” and “Skeletal and muscular disorders” (all *P* < 10^−4^). The top molecular and cellular functions included “Cellular movement”, “Cellular assembly and organization”, “Cellular function and maintenance”, “Cell death and survival” and “Cell-to-cell signalling and interaction” (all *P* < 5 × 10^−4^). The top physiological system development and function included “Digestive system development and function”, “Organismal development”, “Tissue Morphology”, “Nervous system development and function” and “Tissue development” (all *P* < 5 × 10^−4^).

## Discussion

We have found, from conducting two twin and family studies that compared the blood DNA methylation measures between middle-aged BT and AT siblings, evidence consistent with twin birth changing the methylation of siblings born after a twin birth. This change appears to cause the DNA methylation of sibling pairs born after a twin birth to be similar, which is in contrast to the DNA methylation of sibling pairs both before a twin birth, or either side of a twin birth, which is not correlated.

To the best of our knowledge, our studies are the first to report on the potential for a twin birth to influence the methylation of subsequent siblings. The potential for maternal factors to change the methylation of subsequent offspring comes from two studies with similar design that have found that siblings born before and after maternal bariatric surgery have different methylation levels at several genes^14, 15^.

From a previous study that combined data from seven twin and family studies across the lifespan we found evidence that GWAM, a measure of global methylation^16^, is initially determined by intrauterine environmental effects which decrease over the life course (Li, *et al*. Under review). In that study, we did not find evidence that middle-aged sibling pairs overall (including BT, B/AT, AT and twin-sibling pairs) were correlated in GWAM. Here we have found that, on a﻿ closer examination, only the subgroup of AT sibling pairs was correlated. This observation indicates that twin birth can influence the covariance in GWAM for sibling pairs born after a twin birth, even when they are in middle age. It is likely that these pairs were even more highly correlated in earlier life, given that we observed this for twin pairs when we pooled of studies across the life span.

It is unlikely that our observation is explained by factors other than twin birth. Given that both types of siblings are first-degree relatives who share on average 50% of germline genetic information, this observation cannot be explained by such genetic factors. Furthermore, we previously did not find evidence of germline genetic factors explaining variation in GWAM to a detectable extent at any stage of the lifespan (Li, *et al*. Under review). We applied the *ComBat* or a mixed-effects model to minimise batch effects, and we observed similar phenomenon in two independent studies, therefore the observation is unlikely to be due to batch effects, especially those specific to any one study. We did not find evidence that the correlations depended on age, sex or any other factors we examined. It is noteworthy that the mean ages of B/AT pairs were 54.1 and 44.4 years in the AMDTSS and KHTS, respectively, similar to that of AT pairs, yet B/AT pairs were not correlated. We note that our lack of information on maternal age means that we could not examine the influence of this factor on our results. However, given that maternal age is typically negatively correlated with offspring’s age, any impact of maternal age would have been reduced by adjusting for age before estimating the correlations.

Our observations are also unlikely to be explained by the ‘epigenetic drift’, given that the participants were middle-aged. In the seminal paper that first reported this phenomenon, there was little evidence for epigenetic drift in middle age or beyond: middle-aged and elderly pairs of monozygotic twins had similar Euclidean squared distance in 5-methylcytosine content regardless of age^17^. We previously found evidence suggesting that the correlation in GWAM for twin pairs remains constant after early adulthood (Li, *et al*. Under review), which is also consistent with epigenetic drift not being manifest in middle age and beyond.

We found evidence that AT pairs were more correlated in the average methylation of several genomic regions, as well as in methylation at several methylation sites. This suggests that twin birth’s influence is not only detectable at the genome-wide level, but also at specific regions and methylation sites.

Our results are epidemiological observations. Blood methylation has also been observed to be associated with risks of complex traits and diseases; for example, GWAM or similar measures in whole blood have been found to be associated with risks of breast cancer^18, 19^, urothelial cell carcinoma^16^ and mature B-cell neoplasms^20^. The hypothesis of developmental origins of health and disease (DOHaD) considers that epigenome reprogramming during the fetal development period is one possible biological mechanism for the prenatal origins of diseases at later ages^21, 22^. Therefore, our study suggests that siblings born after a twin birth potentially have different disease (e.g. cancer) risks from those born before a twin birth. To our knowledge, no study has yet reported on different disease risks between the two types of siblings defined by a twin birth. Our study also implies that twin birth might mostly influence cell signalling and breast cancer regulation pathways. The overrepresentation of these pathways is mainly based on molecules including *ADCY9*, *ITPR1* and *PRKCZ*. We also find evidence that several disorders/diseases, such as those in connective tissue, development and digestive system, and such cellular functions as cellular movement, assembly and organization involving in tumour cell migration and endocytosis of liposome are potentially influenced by twin birth. The links between these pathways/functions and diseases remain to be investigated.

We hope that our study will provide new insights on the shaping of human blood methylome, especially on the role of intrauterine environment. In addition, our study findings suggest that more research about the features of different types of siblings defined by a twin birth are justified, and have the potential for providing a better understanding the aetiology of complex diseases.

We hypothesize that twin birth influences the DNA methylation of subsequent siblings through changing the intrauterine environment. While our observations suggest that twin birth influences the DNA methylation of subsequent siblings we did not have, and our study could not provide, direct evidence that twin birth changes the intrauterine environment. The mechanisms underlying these epidemiological observations need to be further studied.

A strength of our study is that we have used two independent yet comparable studies for discovery and replication, and found similar results. The inclusion of two studies minimises the possibility for any bias due to factors specific to one study. A limitation of our study is that we were unable to investigate the influence on our results of factors other than those we had measured.

We conclude that twin birth can change the similarity in whole blood DNA methylation of siblings both born after a twin birth, without discernibly changing the methylation level of such siblings.

## Methods

### Subjects

Subjects were from the Australian Mammographic Density Twins and Sisters Study (AMDTSS)^23^ and the Korean Healthy Twin Study (KHTS)^24^. The AMDTSS was used for discovery and the KHTS was used for replication.

The AMDTSS is a twin and family study conducted in Australia originally designed to study mammographic density as a risk factor for breast cancer, in which 479 women comprising 132 twin pairs and their 215 sisters from 130 families were selected for methylation research. The 215 sisters including 112 BT siblings and 103 AT siblings were included in this study. Of the 130 families, 28 included at least two BT siblings, 27 included at least two AT siblings, and 23 included at least one BT sibling and one AT sibling.

The KHTS is a study of twin families conducted in South Korea designed to examine genetic and environmental factors underlying complex human diseases and traits^24^, in which 390 participants comprising monozygotic twins and their first-degree relatives from 97 families were selected for methylation research. 42 siblings including 27 BT siblings and 15 AT siblings were included in this study.

The AMDTSS was approved by the Human Research Ethics Committee of the University of Melbourne. The KHTS was approved by the Institutional Review Board of Samsung Medical Centre and Busan Paik Hospital. Both studies were conducted in accordance with the Helsinki Declaration. All participants from both studies provided written informed consent.

### DNA methylation measurement

Each study measured DNA methylation using the Infinium HumanMethylation450 BeadChip (HM450) array and performed data pre-processing independently.

In the AMDTSS, DNA was extracted from dried blood spots stored on Guthrie cards^25^. DNA was sodium bisulfite converted using the EZ DNA Methylation-Gold protocol as per manufacturers’ instructions (Zymo Research, Irvine, CA) and eluted in 20 µl elution buffer. DNA samples extracted from the same family were assayed on the same chip. Raw intensity data was processed by Bioconductor package *minfi* ^26^ which included normalization of data using Illumina’s reference factor-based normalization methods (*preprocessIllumina*) and subset-quantile within array normalization (*SWAN*)^27^ for type I and II probe bias correction. An empirical Bayes batch-effects removal method *ComBat* ^28^ was applied to minimise the technical variation across batches. All samples passed quality control. Probes with missing value (detection *P*-value > 0.01) in one or more samples, 65 control probes and probes mapping to X-chromosome were excluded, leaving 468,406 autosomal probes. See Li *et al*.^29^ for more details.

In the KHTS, DNA was extracted from peripheral blood lymphocytes. The measurement was conducted in two separated experiments (experiment I and II), with individuals from the same family included in the same experiment. For each experiment, quality control and data pre-processing were performed separately, while the same analytic tools and methods were applied. The R package *RnBeads* ^30^ was applied to extract methylation values. In the quality control, a series of probe and sample filtering steps were followed: probes mapping to sex chromosomes, associated with SNPs and/or out of CpG context were removed, and CpG probes and samples were filtered at detection *P*-value of 0.01. The beta mixture quantile dilation (*BMIQ*) method^31^ was used for normalization.

### Statistical methods

#### Discovery analysis

We studied a global methylation measure, genome-wide average DNA methylation (GWAM) defined as the average beta-value across autosomal probes. We compared the means in GWAM between BT and AT siblings using a linear mixed-effects model, in which GWAM was the outcome and sister type was the predictor. The model was adjusted for age and blood cell type composition (fixed effects), and for family identification number (random effect). The blood cell type composition was estimated from the methylation data using the Houseman method^32^ from the Bioconductor package *minfi*.

We estimated correlations in GWAM for BT pairs (r_BT_), B/AT pairs (r_BAT_) and AT pairs (r_AT_). The correlation was estimated based on computationally maximizing the likelihood of a multivariate normal model for pedigree analysis^33–35^. The correlation in GWAM was the covariance in GWAM between relatives divided by the variance of GWAM. GWAM was firstly adjusted for age and the blood cell type composition using a linear regression. Residuals from the regression were used to estimate correlations. Correlations between BT and AT pairs were compared using the likelihood ratio test (LRT), that is, a nested model in which the two correlations set to equal was fitted, and a *P*-value was calculated according to that twice the difference in the log likelihoods between the full and nested model approximately follows the chi-squared distribution with one degree of freedom.

To investigate if the correlations depend on the twins’ zygosity, we stratified each type of sibling pairs by the twins’ zygosity and estimated the correlation in each stratum. The model fit was compared with that of the model above using the LRT. To investigate if the correlations depend on other factors, we modelled the correlations as α + β *factor where the factor was a characteristic of the sibling pair: (1) the average age; (2) the absolute value of age difference; (3) the average birth order; (4) the absolute value of birth order difference; (5) the average year between the sibling’s birth and the twins’ birth; (6) the absolute value of the difference in year between the sibling’s birth and the twins’ birth; and (7) the twins’ age. The model fit was compared with that of the model above using the LRT.

We studied the average methylation of genomic regions. According to Illumina’s annotation file, probes were grouped according to their genomic positions (gene body, intergenic region, promoter, TSS1500, TSS200, 5′UTR, 1^st^Exon or 3′UTR) or to their positions relative to a CpG island (CGI) (CpG island, CpG shelf, CpG shore or non-CGI region). For each region, the average beta-value across autosomal probes was calculated. The means and correlations of these genomic regions were compared using the same methods as those for GWAM. The binomial test was used to test whether the observed number of statistically significant (at *P* < 0.05) comparisons was more than would be expected by chance.

We studied the methylation measured by each autosomal probe, comparing the means and correlations in the methylation (beta-value) measured by each probe using the same methods as those for GWAM. *P*-values were corrected for the genomic control inflation factor. Genome-wide level of significance was 10^−7^. The McNemar’s test was used to compare the proportions of positive correlation estimates, and the paired *t*-test was used to compare the means in correlation estimates. The binomial test was used to test whether the observed direction of the genome-wide significant associations was more extreme than would have been expected by chance.

### Replication analysis

For GWAM and the average methylation of genomic regions, we estimated their correlations in the KHTS. The methylation measure was firstly adjusted for age, sex, experiment, the estimated blood cell type composition (fixed effects), array and position on the array (random effects) using a linear mixed-effects model in the whole dataset. Residuals from the model were used for correlation estimation. Sex heterogeneity in correlations was examined using the LRT. The binomial test was used to test the consistency of results between discovery and replication analyses.

### Meta-analysis

Results from discovery and replication analyses were pooled using a fixed-effect meta-analysis under inverse-variance weighting. The correlations were compared between BT and AT pairs based on asymptotic theory.

### Pathway analysis

We performed pathway analysis for the probes differentially correlated at *P* < 10^−4^ between BT and AT pairs. These probes were annotated to genes according to the closest transcription start site (TSS)^36^. The gene list was uploaded to QIAGEN Ingenuity^®^ Pathway Analysis (IPA^®^, QIAGEN Redwood City, www.qiagen.com/ingenuity) for assessing overrepresentation relative to all human gene functions^37^.

Correlation estimation and modelling were performed using the Sequential Oligogenic Linkage Analysis Routines (SOLAR) program version 8.1.1 (http://solar-eclipse-genetics.org/). Regression analyses were performed using R version 3.2.4 (http://www.r-project.org). The mixed-effects model was fitted using the *lmer* function from the R package *lme4*. The linear regression was fitted using the *lm* function in R. The meta-analysis was performed using the *metagen* function from the R package *meta*. The McNemar’s test, *t*-test, binomial test were performed using the *mcnemar. test*, *t. test* and *binom. test* function in R, respectively.

### Data availability

The discovery data are available at the Gene Expression Omnibus under accession number GSE100227. The replication data are available on the request to the authors. The SOLAR code could be found in the Supplementary Text.

## Electronic supplementary material

Supplementary Text
