# Supplementary material for: Twin birth changes DNA methylation of subsequent siblings
Source: Sci Rep. 2017 Aug 16;7:8463. doi: 10.1038/s41598-017-08595-6 (PMC5559542; doi:10.1038/s41598-017-08595-6)
Supplement: Supplementary file 1 — Supplementary Text [file 41598_2017_8595_MOESM1_ESM.pdf]

Supplementary Text for

Twin birth changes DNA methylation of subsequent siblings

Shuai Li,[1] Eunae Kim,[2] Ee Ming Wong,[3] Ji-Hoon Eric Joo,[3] Tuong L Nguyen,[1] Jennifer Stone,[4] Yun-Mi Song,[5] Louisa B Flander,[1] Richard Saffery,[6,7] Graham G Giles,[1,8] Melissa C Southey,[3] Joohon Sung,[2,9] and John L Hopper[*1,2,9]

1. Centre for Epidemiology and Biostatistics, Melbourne School of Population and Global Health, The University of Melbourne, Parkville, Victoria, Australia
2. Department of Epidemiology, School of Public Health, Seoul National University, Seoul, South Korea
3. Genetic Epidemiology Laboratory, Department of Pathology, University of Melbourne, Parkville, Victoria, Australia
4. Centre for Genetic Origins of Health and Disease, Curtin University and the University of Western Australia, Perth, Western Australia, Australia
5. Department of Family Medicine, Samsung Medical Center, Sungkyunkwan University School of Medicine, Seoul, South Korea
6. Murdoch Childrens Research Institute, Royal Children's Hospital, Parkville, Victoria, Australia
7. Department of Paediatrics, University of Melbourne, Parkville, Victoria, Australia
8. Cancer Epidemiology Centre, Cancer Council Victoria, Melbourne, Victoria, Australia
9. Institute of Health and Environment, Seoul National University, Seoul, South Korea

Corresponding author: John L Hopper

Address: Centre for Epidemiology and Biostatistics, Melbourne School of Population and Global Health, University of Melbourne, 207 Bouverie Street, Parkville, Victoria 3010, Australia

Email: j.hopper@unimelb.edu.au

Telephone: +61 (03) 8344 0697

SOLAR code

This code is for estimating correlations for pairs of BT, B/AT, and AT siblings in GWAM. GWAM is firstly adjusted for age and cell proportions using a linear regression. The residuals are then standardized to be Z-scores and used in analysis. Two input files, pedigree and phenotype files, are needed. Details of the format of the two files can be found in http://solar-eclipse-genetics.org/. In the phenotype file, variable 'order' indicates the type of sibling, from which '1' means AT sibling, and '2' means BT sibling.

```
## Start of the code

load pedigree ped.ped  #Read pedigree file.

load phenotypes phen.phen #Read phenotype file.

trait gwam #To analyse GWAM (Z-scores for the residuals) from the phenotype file.

parameter rat = 0.0 lower -1.0 upper 1.0 #Define a parameter for the correlation for pairs of
AT siblings, and its initial value and bounds.

parameter rbt = 0.0 lower -1.0 upper 1.0 #Define a parameter for the correlation for pairs of
BT siblings, and its initial value and bounds.

parameter rabt = 0.0 lower -1.0 upper 1.0 #Define a parameter for the correlation for pairs of
B/AT siblings, and its initial value and bounds.

mu = 0.0 #The function of the mean in GWAM.

omega = I + \ #Covariance for pairs of identical individuals.

(phi2 == 0.5)*rat*(order_i == 1)*(order_j == 1) + \ #Covariance for pairs of AT siblings.

(phi2 == 0.5)*rbt*(order_i == 2)*(order_j == 2) + \ #Covariance for pairs of BT siblings.

(phi2 == 0.5)*rabt*(order_i != order_j) #Covariance for pairs of B/AT siblings.

maximize

## End of the code
```
